# Supplementary material for: How to steer active colloids up a vertical wall
Source: Nat Commun. 2024 Feb 24;15:1710. doi: 10.1038/s41467-024-45872-1 (PMC10894264; doi:10.1038/s41467-024-45872-1)
Supplement: Supplementary file 1 — Supplementary Information [file 41467_2024_45872_MOESM1_ESM.pdf]

# Supplementary materials for
# How to steer active colloids up a vertical wall

Adérito Fins Carreira,[1, *] Adam Wysocki,[2, *] Christophe Ybert,[1]
Mathieu Leocmach,[1] Heiko Rieger,[2, 3, †] and Cécile Cottin-Bizonne[1, ‡]

[1] *Université de Lyon, Université Claude Bernard Lyon 1, CNRS, Institut Lumière Matière, F-69622, Villeurbanne, France*
[2] *Department of Theoretical Physics and Center for Biophysics, Saarland University, 66123 Saarbrücken, Germany*
[3] *Leibniz Institute for New Materials INM, Campus D2 2, 66123 Saarbrücken, Germany*

## I. SUPPLEMENTARY METHODS

### A. Measurement of the excess polarity at the wall

#### 1. *Colour shift*

We acquire RGB images with a Nikon water Immersion x60 objective, NA=1.2 and a x1.6 zoom lens. On an image, a pixel is 57 nm ($\approx$ 29 pixels per particle diameter and we have 2048x2048 pixel$^2$. Our color camera (Baumer HGX40c) is composed of a monochrome sensor equipped with colour filter following a Bayer matrix. Therefore there are twice as many green pixels as red or blue pixels. Since the green channel is the most spatially resolved, we use it to localize the particle position with subpixel accuray using the package Trackpy [1]. Around the position of each particle, we localise with subpixel accuracy the local maximum of the red (respectively blue) channel. For each particle, we can thus define two vectors: the 'redshift' vector (respectively 'blueshift' vector) is the difference between the position on the red channel (respectively blue channel) and the position on the green chanel. The results are shown on Fig. S1.

On the left zoom, it is obvious that the redshift vectors are mostly oriented towards the left and the blueshift vectors mostly towards the top right. However on the right zoom the redshift vectors are oriented towards the top right and the blueshift vectors are oriented towards the left. This means that we have chromatic aberration in our optical system: the different wavelength of light are not propagated in the same way, therefore the red image does not form at the same place on the camera as the green image or the blue image. The raw redshift and blueshift vectors must be corrected of this chromatic aberration before they can yield any information on particle polarity.

* These two authors contributed equally
† heiko.rieger@uni-saarland.de
‡ cecile.cottin-bizonne@univ-lyon1.fr

#### 2. *Chromatic aberration*

In order to quantify and correct for the chromatic aberration, we divide the field of view into a $32 \times 32$ grid and average the redshift and blueshift aberration of all the particles that are in a grid element at a given time, for all 6000 images of the video. Since the polarity of chemically bound dimers can be ill defined, we removed all pairs of particles closer than 28px from this ensemble average. Results are shown on Fig. S2.

We want to remove the global chromatic aberration, but not all polarity signal, especially close to the wall. Therefore, we remove from our grid all cells that can contain particles at walls, i.e. what is between the two vertical green dashed lines on Fig. S2. The remaining field is interpolated using splines.

Now, at each instantaneous position of a particle, we can remove from redshift or blueshift the value of the spline at this position. The remaining redshift and blueshift are shown on Fig. S3, this time magnified by a factor 100. The abberation-corrected redshift and blueshift are typically 10 times smaller than the value of the chromatic aberration. Therefore, the arrows are now decorrelated from the apparent color shift on the image.

Many particles have a redshift or blueshift measurement that is below the resolution we can expect from particle localisation from images (0.1 px, here 0.2 px since we subtract two measures). Therefore, we cannot measure precisely the polarity of a given particle, we have to estimate statistical distributions on large numbers of particles. In order to minimze the noise, all the following statistical distributions will be established by weighting a particle contribution by the magnitude of its aberration corrected redshift and/or blueshift.

Furtheremore, by subtracting the average of the redshift or blueshift far from any wall, we removed not only the chromatic aberration, but also any non zero polarity of the bulk. If corrected redshift or blushift contain polarity information, it is the polarity near the wall with respect to the bulk polarity, and not the absolute polarity.

In the following we consider only the abberation-corrected redshift and blueshift and thus drop the adjective.

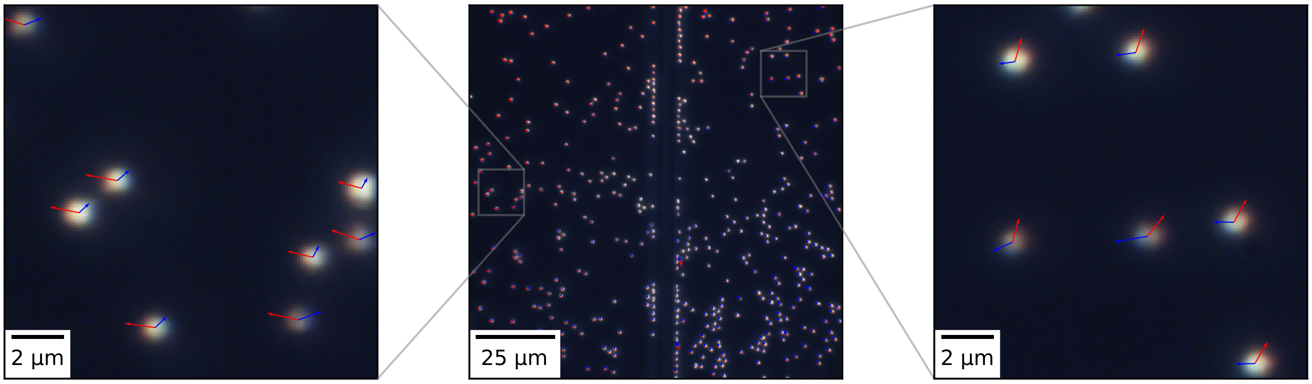


Figure S1. Raw (not corrected for chromatic aberration) redshift and blueshift vectors displayed as red (respectively blue) arrows on top of a white-balanced image. The length of the red and blue arrows are enhanced by a factor 10 for more visibility.

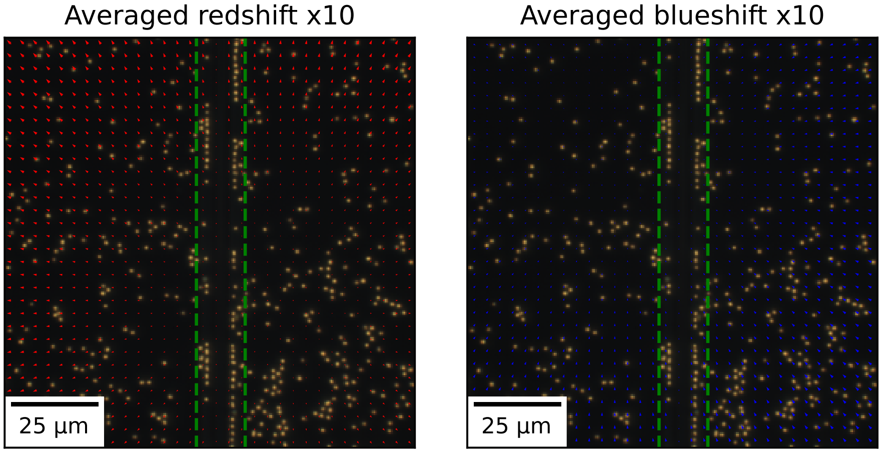


Figure S2. Chromatic aberration observed by averaging redshift and blueshift on a grid and in time. The length of the red and blue arrows are enhanced by a factor 10 for more visibility. The vertical green line delimit the region of the wall.

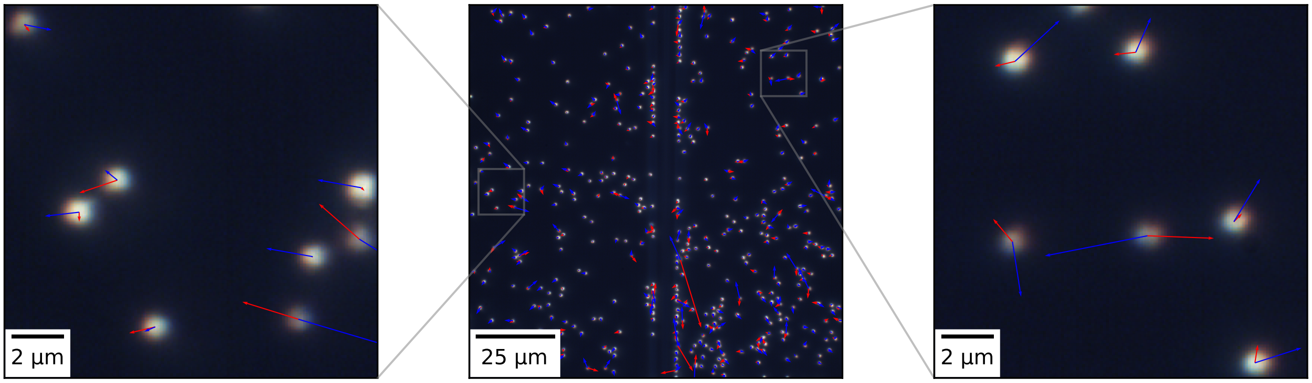


Figure S3. Chromatic aberration-corrected redshift and blueshift vectors displayed as red (respectively blue) arrows on top of a white-balanced image. The length of the red and blue arrows are enhanced by a factor 100 for more visibility.

### 3. Statistical correlation between colour shift and polarity

First, we can compute the probability distribution function of the angle $\theta_i^{\text{red blue}}$ between redshift $\overrightarrow{S_i^{\text{red}}}$ and blueshift $\overrightarrow{S_i^{\text{blue}}}$ of particle $i$:

$$P^{\text{red blue}}(\theta) = \frac{\sum_i S_i^{\text{red}} S_i^{\text{blue}} \delta(\theta_i^{\text{red blue}} - \theta)}{\sum_i S_i^{\text{red}} S_i^{\text{blue}}}, \quad (1)$$

where $\delta$ is the binning function, $S_i^{\text{red}} = \left\| \overrightarrow{S_i^{\text{red}}} \right\|$ and dimers are excluded from the sums. Results are shown on Fig. S4 (Left).

Redshift and blushift are statistically antiparallel in bulk, but the peak is quite large. At wall, they are cleany split between two populations, either antiparallel (majority) or parallel (minority).

Then, we compute the probability distribution function of the angle $\theta_i^{\text{red}}(t)$ between redshift at time $t$ and the displacement $\overrightarrow{\Delta x}(t)$ of the particle $i$ between $t$ and $t + \delta t$, where $\delta t = 100\,\text{ms}$ is the time interval between two successive frames:

$$P^{\text{red}}(\theta) = \frac{\sum_t \sum_i S_i^{\text{red}} \delta(\theta_i^{\text{red}} - \theta)}{\sum_t \sum_i S_i^{\text{red}}}, \quad (2)$$

where sums exclude dimers and particles at the wall. We define $P^{\text{blue}}(\theta)$ in the same way. Both distributions are shown on Fig. S4 (Right).

As $P^{\text{red}}(\theta)$ is flat, the redshift is not correlated to displacement and is thus not a measure of polarity. By contrast, $P^{\text{blue}}(\theta)$ shows a peak around $\pi$, meaning that blue shift is mostly antiparallel to displacement.

Even far from the walls, particle displacement is an indirect measure of polarity, as particles experience forces other than self-propulsion, i.e. Brownian motion, gravity and particle-particle interactions of electrostatic and hydrodynamic nature. In addition, automatic trajectory reconstruction is subject to tracking errors, which further decrease the signal-to-noise ratio. Despite these limitations, blueshift is a good *statistical* predictor of particle displacement and polarity.

### 4. Rationale of blueshift

*a. Why is blueshift a good measure of polarity and not redshift?* The platinum cap is thin, thus the light reflected by the particles is mostly yellow, that is to say a mix of red and green with much less blue. The difference between red position and green position (redshift) thus contains little information. By contrast, platinum reflects more equally the colors, therefore the excess of blue with respect to green (or red) indicates the platinum side.

*b. But why are redshift and blueshift antiparallel?* The filters on a colour camera slightly overlap. In particular the green channels also captures some red on one side and some blue on the otherside, whereas there is very little cross-talk between red and blue channels. With respect to the red position, the green position is thus slightly shifted towards the blue position. This shift, is enough to cause the statistical correlation between redshift and blueshift, but the signal is so small with respect to noise that the redshift is a poor predictor of polarity and even poorer predictor of the direction of motion.

## B. Wall interaction for Janus self-phoretic particles

We discuss hereafter the form and magnitude of the wall–particle interaction used in numerical simulations.

### 1. Form of the potential

The modelling of self-phoretic swimmers is a complex problem [2–4], where even the mere sign of interactions –attractive vs repulsive– and the presence or not of alignment depends on tiny physicochemical details of the particles that we cannot measure directly. Instead of this first-principle approach, we chose to use a simple potential that allows us to understand the basic ingredients that rule the experimental system behaviour. To account for a possible attraction we use a Lennard-Jones potential of depth $\epsilon$, whereas the possible bipolar alignment is ruled by a position-dependent torque of strength $\Gamma$.

In addition to the Lennard-Jones potential we also checked another standard potential used in computational models of active colloids, i.e. the Yukawa potential, and did not find significant differences in the observables that we measured (see Fig. S5). This is actually expected since both interactions are very short-ranged.

More precisely, we used an effective Yukawa potential of the form

$$V_{Yukawa}(x) = -Y \frac{e^{-\kappa x}}{x} \quad (3)$$

to account for attractive phoretic (far-field) wall-particle interactions. The steric repulsion between the wall and the particles was modeled via a Lennard-Jones potential truncated at $x/R = 2^{1/6}$. We fixed $\kappa R = 2$ and adjusted the strength of attraction $Y$ such that the second virial coefficient $B_2$ of the potentials to be compared is equal.

### 2. Diffusiophoretic wall attraction

Gold-Platinum Janus particles have been shown to experience an activity-induced adhesive interaction of phoretic origin [5]. Indeed each catalytic particle act as a chemical monopole source from the far-field creating environmental gradients to which each swimmer responds. One thus expects that such attractive interaction is experimentally related to the phoretic mechanism responsible for the self-propulsion for which the associated swim

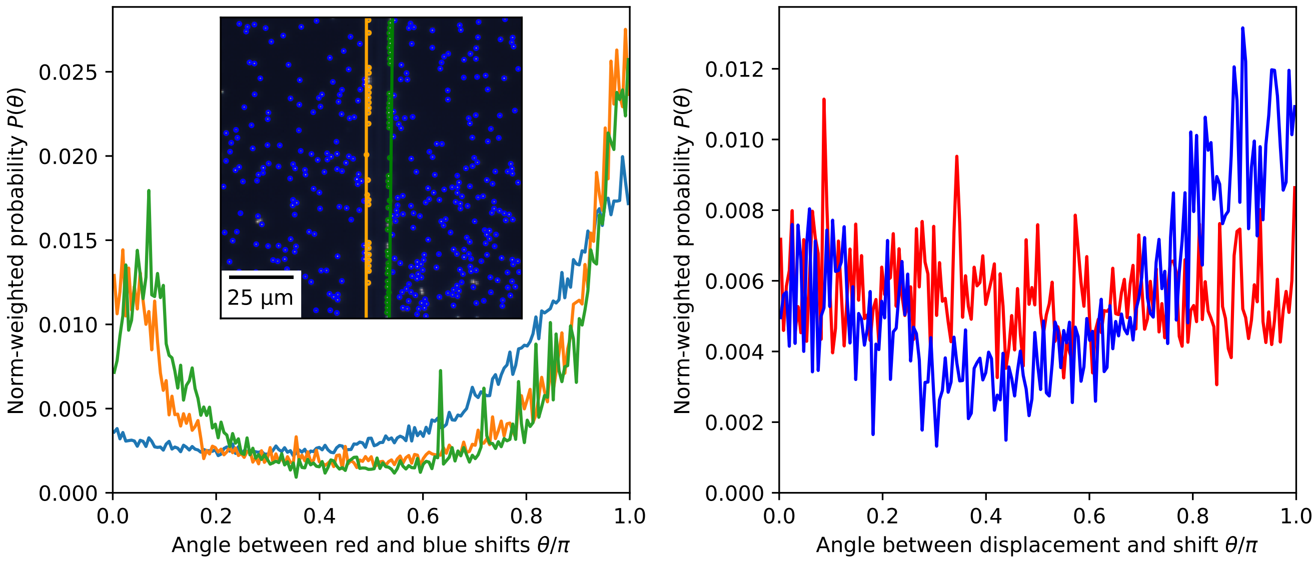


Figure S4. Left: Probability distribution of the angle between abberation-corrected redshift and blueshift of the same particle. Colors correspond to particles in bulk, left wall and right wall, as indicated on the inset. Right: Probability distribution of the angle between the displacement of a particle between $t$ and $t+\delta t$ and the abberation-corrected redshift (respectively blueshift) of this particle at time $t$ averaged over particles in bulk and time $t$.

force reads

$$F_{\rm swim} = \frac{k_B T_0}{D_t} v_0. \tag{4}$$

Setting that the diffusiophoretic adhesion with the wall therefore involves an adhesion energy $\epsilon_{\rm phor.} \sim F_{\rm swim} R$, we thus expect an adhesion of dimensionless form

$$\widetilde{\epsilon}_{\rm phor.} = \frac{3}{4}{\rm Pe_s}, \tag{5}$$

where as in the main text $\widetilde{\epsilon}_{\rm phor.} = \epsilon_{\rm phor.}/k_B T_0$ and ${\rm Pe_s} = v_0/(R D_r)$.

Overall, this justifies the form used in numerical simulation for the wall adhesion $\widetilde{\epsilon} = \alpha {\rm Pe_s}$.

### *3. Wall aligning interaction*

As stated in the main text, the aligning torque was taken based on the effect of hydrodynamic interactions between the wall and a pusher swimmer [6]. Indeed this was argued to be the dominant contribution for phoretic Janus particles [7] with an associated torque magnitude $t$ reading for small deviations from wall alignment

$$t = k_B T_0 \left(\frac{R}{R+h}\right)^3 (2\Delta\theta) \times \frac{1}{8}{\rm Pe_s}, \tag{6}$$

As compared to the form adopted in simulations, this amounts to a dimensionless torque magnitude of $\widetilde{\Gamma}_{\rm hydro.} = \frac{1}{8}{\rm Pe_s}$. Again, this motivated the form $\widetilde{\Gamma} = \beta {\rm Pe_s}$.

When considering pure particle wall interactions (either attractive or aligning), magnitude $\alpha$ or $\beta$ were chosen according in the range suggested by the previous rough estimations: ($\alpha = 0.5, \beta = 0.5$) When considering a combination of wall attraction and wall-aligning interactions in the numerical model, we adjusted the parameters to achieve a better match with the experiment. To find the best parameters, we utilized our ABP model to construct a map of the detention time $\tau_{detention}$ and wetting height $\Delta H$ as a function of wall-attraction $\tilde{\epsilon}$ and wall-alignment $\tilde{\Gamma}$ for ${\rm Pe_s} = 13$ (Supplementary Figure S6) and found a good match, within our model, to the experimental data for $\tilde{\epsilon} = 0.25{\rm Pe_s}$ and $\tilde{\Gamma} = 2{\rm Pe_s}$ (cross in both panels of Supplementary Figure S6). Considering how much this ABP model simplifies the complexity of phoretic swimmers and their wall-interactions, these magnitudes are in quite fair agreement with the previous rough estimations. A more quantitative match, especially for the aligning interactions which appears stronger-than-expected in the numerical simulations, would require a more refined and complex approach tackling the details of the wall-swimmer physics.

## C. Fluxes and wall dynamics

To obtain the circulating steady-state currents numerically and reduce noise, the data are averaged over a time of $10^6\tau_r$, which, unfortunately, is not achievable in the experiments. In the experiments, we were able to get a good signal-to-noise ration on the vertical flow only in the dense part of the adhesion layer. In this region, we

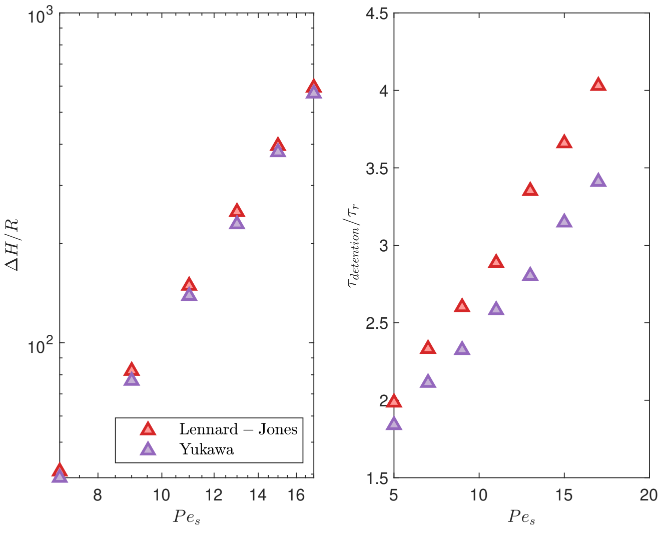


Figure S5. Effect of the form of the wall-particle potential on adsorption layer height $\Delta H(\mathrm{Pe_s})$ and detention time $\tau_{detention}(\mathrm{Pe_s})$. We compare an untruncated Lennard-Jones potential of depth $\epsilon$ (repulsive for $x/R < 2^{1/6}$ and attractive for $x/R > 2^{1/6}$) to an attractive Yukawa potential (steric repulsion is modelled via a purely repulsive Lennard-Jones potential truncated at $x/R = 2^{1/6}$). We adjusted the strength $Y$ of the Yukawa potential such that the second virial coefficient $B_2$ of both potentials is equal. Here we used exemplary $\tilde{\epsilon} = 0.5\mathrm{Pe_s}$ for the adhesion and $\tilde{\Gamma} = 0.5\mathrm{Pe_s}$ for the alignment strength.

observe a negative flux, corresponding to downward flow. This corresponds to the downward flow measured numerically, although with a lower amplitude (see Figure S7).

The numerical model also exhibits trains and we also observe that their distribution decays exponentially see figure S8 and see Supplementary movies `Pe_17_epsilon_4.25_Gamma_34.avi`. The deviation from pure exponential law at small sizes can be explained by gravity effects, not taken into account in the simple model of the main text.

## II. SUPPLEMENTARY NOTES

### A. Description of Additional Supplementary Files

**Supplementary Movie 1:** Sediment of active Janus colloids at $\mathrm{Pe_s} = 13.7$ near the vertical wall on the left, for $z \in [-76; 368]$ µm. The movie was sped up twice.
`71T0_x2_overview.avi`

**Supplementary Movie 2:** Close up near the vertical wall of active Janus colloids at $\mathrm{Pe_s} = 13.7$ for $z \in [60; 281]$ µm. The movie was sped up twice.
`71T0_x2_zoom.avi`

**Supplementary Movie 3:** Numercial sediment of ABP near the vertical wall on the left ($x = 0$) at activity $\mathrm{Pe_s} = 17$, alignment $\tilde{\Gamma} = 2\mathrm{Pe_s}$ and adhesion strength $\tilde{\epsilon} = 0.25\mathrm{Pe_s}$. The left part is a close up of the right part indicated by the green box.
`Pe_17_epsilon_4.25_Gamma_34.avi`

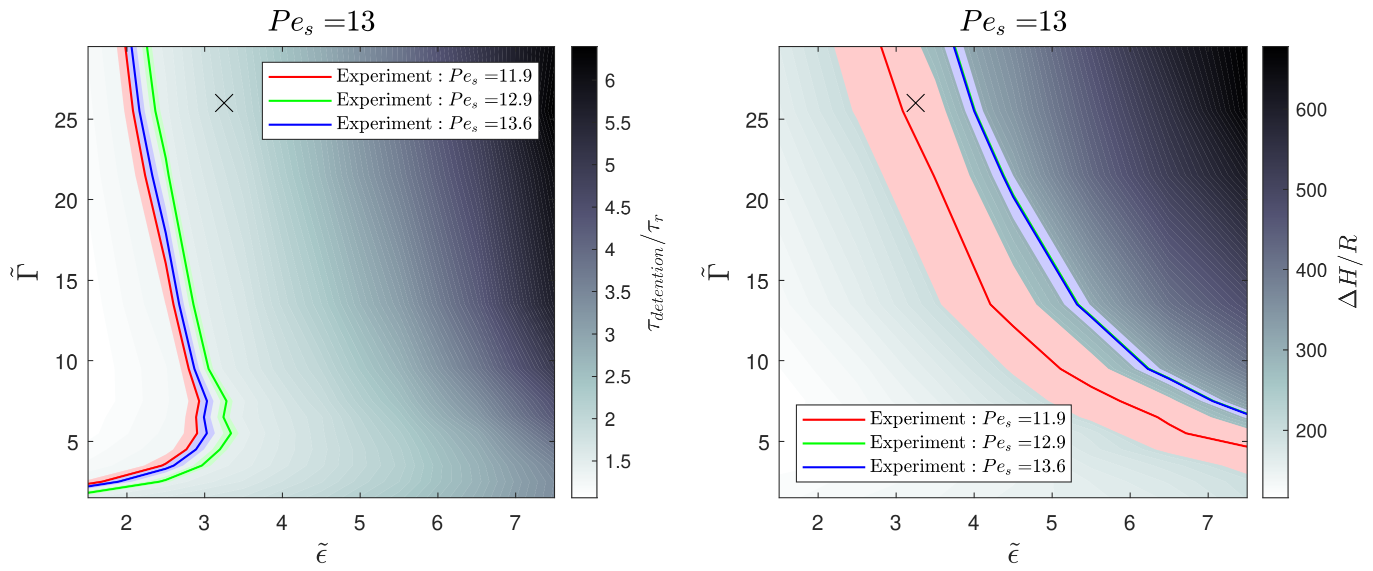


Figure S6. Map of the detention time $\tau_{detention}$ (left) and wetting height $\Delta H$ (right) as a function of wall-attraction $\tilde{\epsilon}$ and wall-alignment $\tilde{\Gamma}$ for our ABP model and for $\mathrm{Pe_s} = 13$. The isolines are at $\tau_{detention}$ and $\Delta H$ obtained from the experiment at comparable values of the activity $\mathrm{Pe_s} = 11.9,\ 12.9,\ 13.6$. The shade around the isolines indicates the error of $\tau_{detention}$ and $\Delta H$. The cross indicates the parameters used in the manuscript.

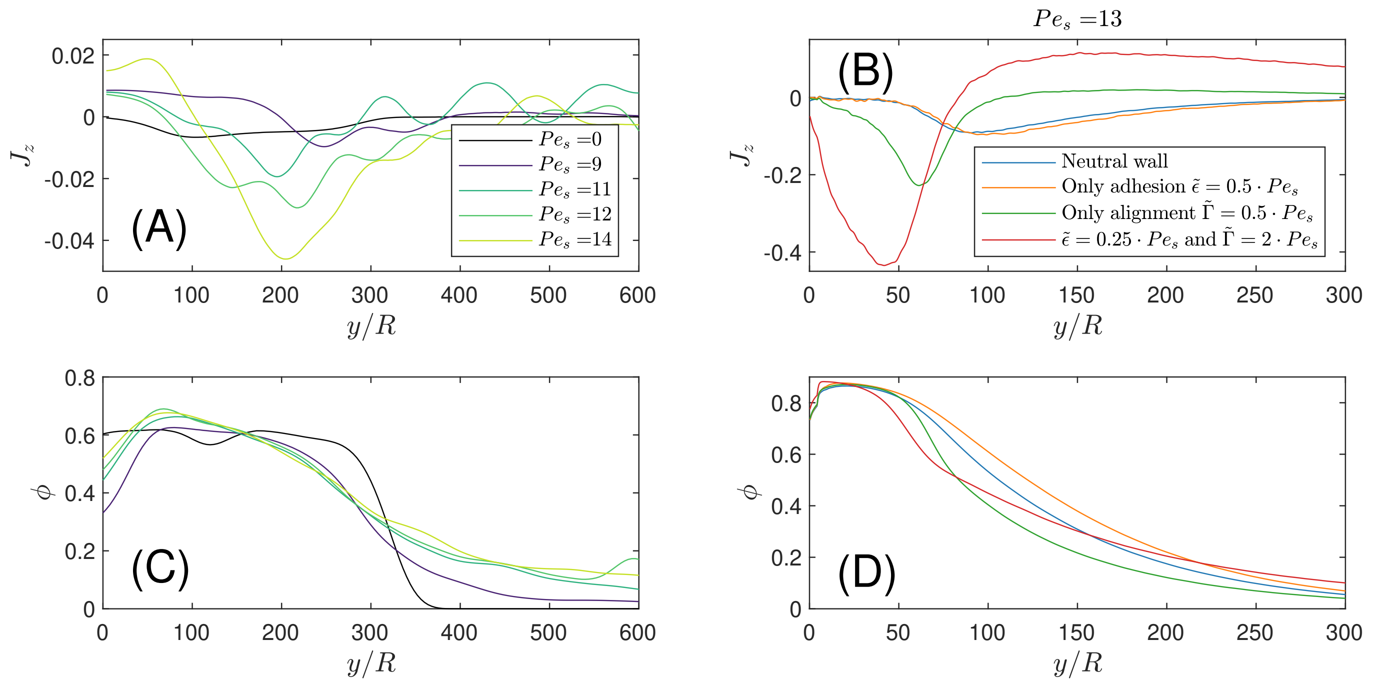


Figure S7. Fluxes (A and B) and density profiles (C and D) at the wall. Experimental (A and C) and numerical (B and D) measurements.

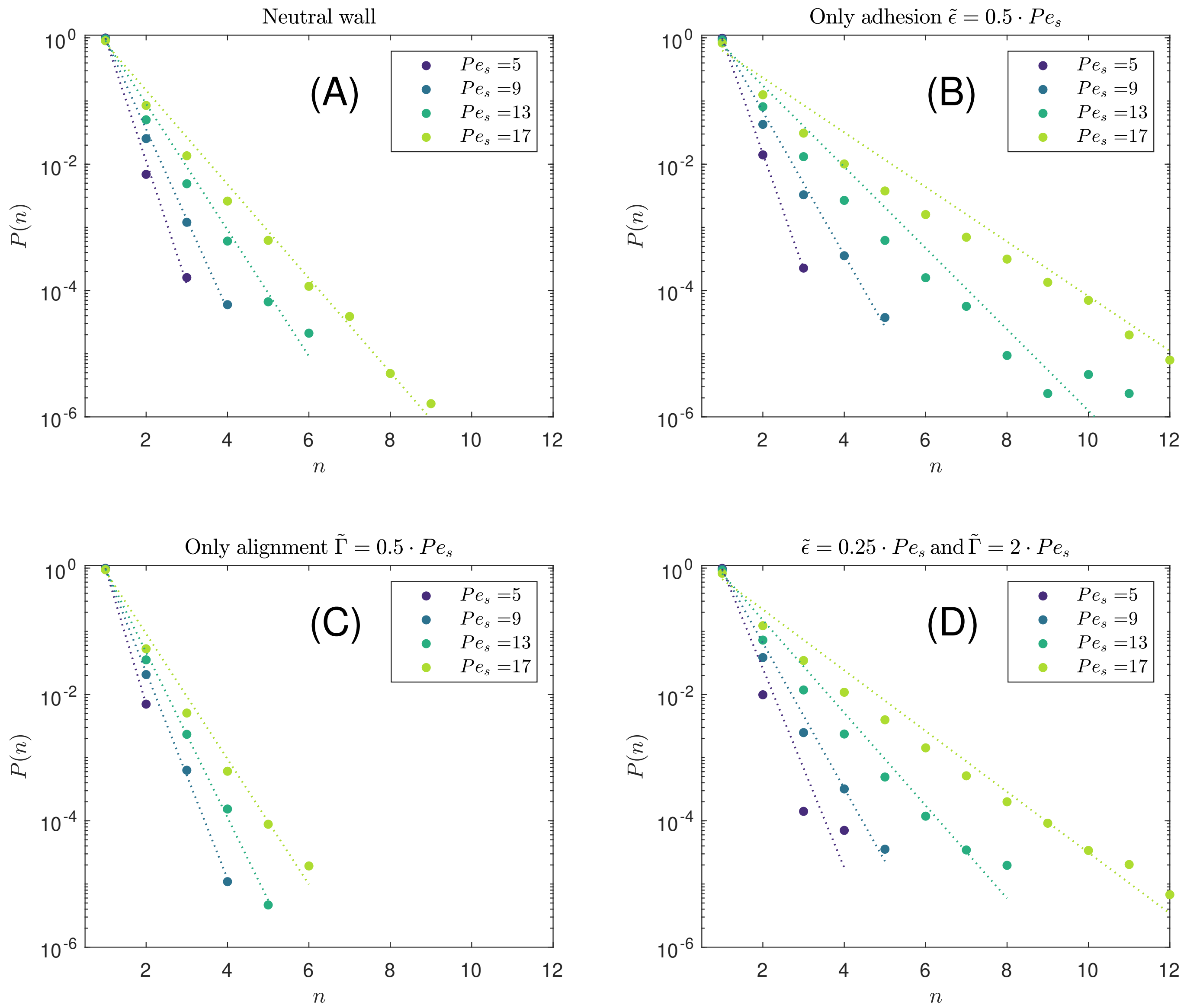


Figure S8. Trains statistics in the ABP model. Probabilities of the train size in the adsorption layer $P(n)$ for different wall-particles interactions. (A): Neutral wall, (B): only adhesion (($\tilde{\epsilon} = 0.5\mathrm{Pe_s}$), (C): only alignment ($\tilde{\Gamma} = 0.5\mathrm{Pe_s}$) and (D): adhesion and alignment ($\tilde{\epsilon} = 0.25\mathrm{Pe_s}$ and $\tilde{\Gamma} = 2\mathrm{Pe_s}$). Dotted lines represent a geometric law fit to the data.

## III. SUPPLEMENTARY REFERENCES


[1] D. Allan, T. Caswell, N. Keim, and C. van der Wel, trackpy: Trackpy v0.3.2 (2016).

[2] Y. Ibrahim and T. B. Liverpool, Eur. Phys. J. Special Topics **225**, 1843 (2016).

[3] E. Kanso and S. Michelin, The Journal of Chemical Physics **150**, 044902 (2019).

[4] R. Singh, R. Adhikari, and M. E. Cates, The Journal of Chemical Physics **151**, 044901 (2019).

[5] F. Ginot, I. Theurkauff, D. Levis, C. Ybert, L. Bocquet, L. Berthier, and C. Cottin-Bizonne, Physical Review X **5**, 011004 (2015).

[6] A. P. Berke, L. Turner, H. C. Berg, and E. Lauga, Physical Review Letters **101**, 038102 (2008).

[7] S. Das, A. Garg, A. I. Campbell, J. Howse, A. Sen, D. Velegol, R. Golestanian, and S. J. Ebbens, Nature Communications **6** (2015).
