# Supplementary material for: How to steer active colloids up a vertical wall
Source: Nat Commun. 2024 Feb 24;15:1710. doi: 10.1038/s41467-024-45872-1 (PMC10894264; doi:10.1038/s41467-024-45872-1)
Supplement: Supplementary file 3 — Description of Additional Supplementary Files [file 41467_2024_45872_MOESM3_ESM.docx]

**Description of Additional Supplementary Files**

**File Name: Supplementary Video 1
Description:** Sediment of active Janus colloids at Pes = 13.7 near the vertical wall on the left, for z ∈ [−76; 368] µm. The movie was sped up twice. 71T0 x2 overview.avi

**File Name: Supplementary Video 2
Description:** Close up near the vertical wall of active Janus colloids at Pes = 13.7 for z ∈ [60; 281] µm. The movie was sped up twice. 71T0 x2 zoom.avi

**File Name: Supplementary Video 3
Description:** Numercial sediment of ABP near the vertical wall on the left (x = 0) at activity.
